# Supplementary material for: Active site specificity profiling datasets of matrix metalloproteinases (MMPs) 1, 2, 3, 7, 8, 9, 12, 13 and 14
Source: Data Brief. 2016 Feb 22;7:299–310. doi: 10.1016/j.dib.2016.02.036 (PMC4777984; doi:10.1016/j.dib.2016.02.036)
Supplement: Supplementary file 10 — Supplementary material [file mmc10.zip › WebPICS_hMMP7_G_1%/P3prime.html]

 

PICS results


|  |  |
| --- | --- |
| **P3prime\_A**  28 in 163 sites   17.2 %    effects > 10 perc. pnts.   (vice-versa in brackets)  P3\_I: 17.6 (41.1)   P2\_Q: 11.2 (28.3) |  |
| **P3prime\_C**  3 in 163 sites   1.8 %    effects > 10 perc. pnts.   (vice-versa in brackets)  P2\_C: 30.8 (23.2) |  |
| **P3prime\_G**  19 in 163 sites   11.7 %    effects > 10 perc. pnts.   (vice-versa in brackets)  P1\_R: 25.8 (27.2)   P2prime\_I: 16.5 (19.5) |  |
| **P3prime\_K**  25 in 163 sites   15.3 %    effects > 10 perc. pnts.   (vice-versa in brackets)  P2\_A: 14.5 (16.5) |  |
| **P3prime\_N**  8 in 163 sites   4.9 %    effects > 10 perc. pnts.   (vice-versa in brackets)  P1\_N: 17.6 (11.8) |  |
| **P3prime\_Q**  11 in 163 sites   6.7 %    effects > 10 perc. pnts.   (vice-versa in brackets)  P1\_K: 16.9 (10.9)   P1prime\_M: 13.3 (18.3)   P2prime\_R: 18.1 (13.3) |  |
| **P3prime\_S**  11 in 163 sites   6.7 %    effects > 10 perc. pnts.   (vice-versa in brackets)  P2\_K: 18.7 (14.7)   P1\_G: 19.9 (18.3) |  |
| **P3prime\_V**  13 in 163 sites   8.0 %    effects > 10 perc. pnts.   (vice-versa in brackets)  P3\_P: 13.3 (10.8)   P2\_K: 14.5 (13.4)   P2prime\_Q: 16.4 (19.3)   P2prime\_T: 13.3 (10.8) |  |
